# Mapping of positive selection sites in the HIV-1 genome in the context of RNA and protein structural constraints

**DOI:** 10.1186/1742-4690-8-87

**Published:** 2011-11-01

**Authors:** Joke Snoeck, Jacques Fellay, István Bartha, Daniel C Douek, Amalio Telenti

**Affiliations:** 1 Institute of Microbiology, University Hospital Center and University of Lausanne, Lausanne, Switzerland; 2 Rega Institute for Medical Research, KU Leuven, Leuven, Belgium; 3 Global Health Institute, School of Life Sciences, EPFL, Lausanne, Switzerland; 4 Eötvös Lorand University, Institue of Biology, Budapest; 5 Human Immunology Section, Vaccine Research Center, National Institute of Allergy and Infectious Diseases, NIH, Bethesda, Maryland, USA

**Keywords:** HIV, evolution, positive selection, RNA structure

## Abstract

**Background:**

The HIV-1 genome is subject to pressures that target the virus resulting in escape and adaptation. On the other hand, there is a requirement for sequence conservation because of functional and structural constraints. Mapping the sites of selective pressure and conservation on the viral genome generates a reference for understanding the limits to viral escape, and can serve as a template for the discovery of sites of genetic conflict with known or unknown host proteins.

**Results:**

To build a thorough evolutionary, functional and structural map of the HIV-1 genome, complete subtype B sequences were obtained from the Los Alamos database. We mapped sites under positive selective pressure, amino acid conservation, protein and RNA structure, overlapping coding frames, CD8 T cell, CD4 T cell and antibody epitopes, and sites enriched in AG and AA dinucleotide motives. Globally, 33% of amino acid positions were found to be variable and 12% of the genome was under positive selection. Because interrelated constraining and diversifying forces shape the viral genome, we included the variables from both classes of pressure in a multivariate model to predict conservation or positive selection: structured RNA and α-helix domains independently predicted conservation while CD4 T cell and antibody epitopes were associated with positive selection.

**Conclusions:**

The global map of the viral genome contains positive selected sites that are not in canonical CD8 T cell, CD4 T cell or antibody epitopes; thus, it identifies a class of residues that may be targeted by other host selective pressures. Overall, RNA structure represents the strongest determinant of HIV-1 conservation. These data can inform the combined analysis of host and viral genetic information.

## Background

The HIV-1 genome is highly polymorphic, for several reasons. Firstly, different cross-species transmission events gave rise to different viral lineages in humans [1]. In addition, intrinsic characteristics of the virus, such as its short generation time, and lack of proofreading activity of the reverse transcriptase further increase genetic variability [2]. The virus is capable of genomic recombination, and most of its proteins tolerate coding variation [3,4]. Based on this genetic diversity, HIV-1 can be classified into several types, groups, and subtypes [5].

Theoretically, every single mutation at every position in the genome is generated every day. However, most of the resulting virions are not viable, and various layers of conservation (RNA and protein structure and use of overlapping coding frames) may effectively constrain the level of genomic variability [6,7]. On the other hand, there are recognized pressures that target the virus resulting in escape and adaptation (pressure exerted by the immune system or by antiviral treatment and bottleneck events such as transmission) [8,9]. These opposing forces need to be considered for a correct understanding of evolution of the viral genome.

Here we aim at generating a comprehensive map of the HIV-1 genome including information on conservation, positive selective pressure, and structural constraints, which has the potential to serve as a reference for understanding the limits to viral escape, and for the discovery of sites of genetic conflict between viral and host proteins.

## Results

### The nature of the conserved genome

**Figure 1:**
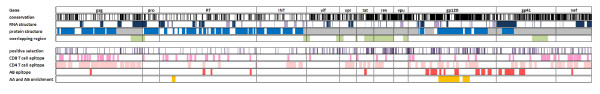
**Map of the HIV-1 genome**. For clarity, the genome is represented as linear, with the genes represented as a concatemer (top bar). The following layers of data are shown: **Conservation: **black = amino acid conservation less than 95%. **RNA**: dark blue = extensively structured RNA (SHAPE parameter < 0.25), light purple = flexible RNA (SHAPE > 0.5). **Protein structure**: blue = structured (β-sheet or α-helix), grey = no structural information available for *vif*, *vpr*, *tat*, *rev*, *vpu *and *nef*. **Overlapping region**: green = sites in overlapping reading frames. **Positive selection**: dark purple = sites under positive selection. **CD8 T cell epitope**: pink = CD8 T cell epitope, **CD4 T cell epitope**: light pink = CD4 T cell epitope, **AB epitope **: red = antibody epitope, **AA and AG enrichment**: orange = regions enriched in AA and AG dinucleotide motives.

Although the HIV-1 genome is highly variable, 67% of amino acid positions were found to be conserved. We built a map of the genome that shows the association between conservation and structural constraints: protein and RNA structure, and presence of overlapping coding frames (Figure 1). Seventy-two percent of residues in structured RNA regions and 74% of residues in structured protein regions (67% in β-sheets and 81% in α-helices) were conserved. The need to use overlapping reading frames is generally thought to be another level of constraint of viral diversity. However, in this dataset, only over half of the residues in these regions were conserved (56%). Univariate statistical analyses (Table 1, **section A**) indicated that RNA secondary structure and α-helix domains effectively limit viral variability (OR 1.29 (1.05-1.6), p = 0.02 and 1.52 (1.17-1.98), p = 0.002, respectively). In contrast, β-sheet domains and overlapping reading frames showed increased levels of variability (OR 0.74 (0.56-0.97), p = 0.03; and OR 0.55 (0.45-0.68), p = 7.3E-09, respectively).

**Table 1:** Result of the univariate statistics for association with (A) conservation, or (B) positive selection.

(A) Univariate - conservation																										
	
	Genome		gag		pro		rt		int		vif		vpr		tat		rev		vpu		gp120		gp41		nef	
	OR (95% CI)	p	OR (95% CI)	p	OR (95% CI)	p	OR (95% CI)	p	OR (95% CI)	p	OR (95% CI)	p	OR (95% CI)	p	OR (95% CI)	p	OR (95% CI)	p	OR (95% CI)	p	OR (95% CI)	p	OR (95% CI)	p	OR (95% CI)	p
	
Flexible RNA regions	0.93 (0.69-1.26)	NS		na		na	1.27 (0.55-3.45)	NS	1.38 (0.58-3.82)	NS	0.92 (0.27-3.62)	NS		na	5.42E+06(8.53E-123-∞)	NS	5.65E+06 (8.53E-123-∞)	NS	2.5 (0.86-7.47)	NS	0.58 (0.33-0.99)	0.05	1.69 (0.16-3.66)	NS	0.58 (0.074-2.12)	NS
Structured RNA regions	1.29 (1.05-1.6)	0.02	0.54 (0.34-0.87)	0.01	1.05 (0.37-3.23)	NS	3.19 (1.46-8.43)	0.008		na	0.58 (0.23-1.46)	NS	1.07 (0.38-3.31)	NS	1.63E-07(∞-1.04E+122)	NS	0.98 (0.18-5.51)	NS	3.47E-07(∞-2.13E+122)	NS	1.73 (0.93-3.31)	NS	2.87 (1.75-4.82)	4E-05	1.19 (0.67-2.12)	NS
α-helix structures	1.52 (1.17-1.98)	0.002	2 (1.17-3.42)	0.01	1.06 (0.14-2.20)	NS	0.9 (0.55-1.49)	NS	0.9 (0.42-1.9)	NS		na		na		na		na		na	1.1 (0.57-2.11)	NS	3.93 (0.48-2.45)	NS		na
β-sheet structures	0.74 (0.56-0.97)	0.03		na	0.38 (0.12-1.11)	NS	1.14 (0.63-2.14)	NS	1.94 (0.7-6.24)	NS		na		na		na		na		na	1.05 (0.66-1.66)	NS		na		na
Overlapping regions	0.55 (0.45-0.68)	7.3E-09	0.51 (0.3-0.89)	0.02	0.63 (0.18-2.5)	NS		na	2.78 (0.55-50.9)	NS	1.55 (0.74-3.48)	NS	0.84 (0.32-2.33	NS	0.25 (0.1-0.58)	0.002		na	1.10 (0.42-2.8)	NS		Na	0.53 (0.33-0.86)	0.01		na
**(B) Univariate - positive selection**																										
	
	**Genome**		**gag**		**pro**		**rt**		**int**		**vif**		**vpr**		**tat**		**rev**		**vpu**		**gp120**		**gp41**		**nef**	
	OR (95% CI)	**p**	OR (95% CI)	**p**	OR (95% CI)	**p**	OR (95% CI)	**p**	OR (95% CI)	**p**	OR (95% CI)	**p**	OR (95% CI)	**p**	OR (95% CI)	**p**	OR (95% CI)	**p**	OR (95% CI)	**p**	OR (95% CI)	**p**	OR (95% CI)	**p**	OR (95% CI)	**p**
	
CD8 T cell epitope	0.85(0.64-1.12	NS	**0.4 (0.17-0.83)**	**0.02**	1.21E-07(∞-1.03E+88)	NS	0.69 (0.2-1.85)	NS	2.46(0.13-1.48)	NS	1.44 (0.21-6.11)	NS	1.23E-07(∞-8.03E+38)	NS	0.29 (0.015-1.68)	NS	1.22 (0.36-3.599	Ns		na	**2.66 (1.5-4.63)**	**0.0006**	0.76 (0.36-1.52)	NS	0.84 (0.35-1.95)	NS
CD4 T cell epitope	1.03 (0.82-1.28)	NS	0.84(0.42-1.79)	NS		na	0.81 (0.13-2.86)	NS	1.06 (0.28-3.37)	NS		na	**0.27 (0.069-0.88)**	**0.04**	**0.25 (0.083-0.66)**	**0.007**	**0.075 (0.011-0.27)**	**6.96E-04**		na	1.54 (0.99-2.37)	NS	**2.13 (1.16-3.89)**	**0.01**	0.66 (0.28-1.59)	NS
AB epitope	**1.73 (1.28-2.32)**	**0.0005**	6.14E-07(∞-1.78E+15)	NS		na	0.62 (0.034-3.09)	NS		na		na		na	0.44 (0.065-1.76)	NS		na		na	**1.6 (1.02-2.49)**	**0.04**	0.87 (0.44-1.64)	NS	3.98 (0.8-162)	NS

The odds ratio (95% confidence interval) and the p-value are shown for the entire genome and for each gene separately. Results in bold are statistically significant. NS: not significant, na: not applicable

We also performed a similar analysis for each gene separately. Secondary RNA structure played variable roles depending on the HIV-1 genes: structured RNA regions were associated with conservation for *RT *and *gp41*, but with variability in *gag*, while flexible RNA regions were associated with increased variability in *gp120*. Regarding protein structure, only α-helix domains in *gag *were associated with conservation; no association was found for β-sheets. Overlapping reading frames were associated with more variability in *gag*, *tat *and *gp41*. However, some of the gene-level subanalyses were limited by the length of the gene or the number of informative sites, and thus by the statistical power.

### The nature of genomic regions under positive selective pressure

Evolutionary pressures are strong driving forces for viral diversity. Twelve percent of the viral genome were found to be under positive selective pressure in the study dataset. It is believed that selective pressure predominantly acts on viral epitopes that are targets for the host immune system. We therefore mapped sites under positive selection, together with sites within known CD8 T cell, CD4 T cell, and antibody epitopes on the HIV-1 genome (Figure 1). We found that 11% of residues in CD8 T cell epitopes, 13% of residues in CD4 T cell epitopes and 18% of residues in antibody epitopes were under positive selection. Univariate analysis showed a significant association between sites under positive selection and antibody epitopes (OR 1.73 (1.28-2.32), p = 0.0005), but not CD8 or CD4 T cell epitopes (Table 1, **section B**).

Analyses were also performed for each gene separately. We confirmed the association of sites under positive selection and antibody epitopes for *gp120*. While associations with CD8 T cell and CD4 T cell epitopes were not significant in the genome-wide analysis, we did find significant associations for specific genes: associations with positive selection were observed in *gp41 *for CD4 T cell epitopes, and in *gag *and *gp120 *for CD8 T cell epitopes.

### Combining different layers of data

**Figure 2:**
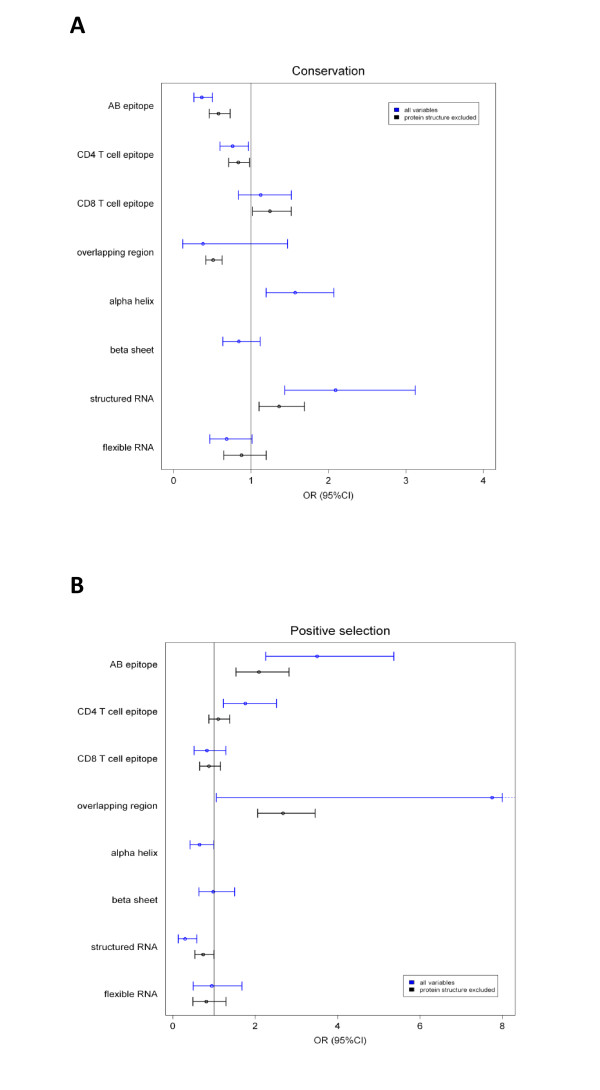
**Multivariate analysis of constraining and diversifying forces shaping the viral genome**. For each variable, the odds ratio and 95% confidence interval are shown for a multivariate model predicting conservation (A) or positive selection (B). The vertical line shows the null hypothesis (OR = 1). Two separate analyses were performed, one including all variables (blue) and one excluding protein structure, not available for 45% of the genome (black). OR = odds ratio, CI = confidence interval.

Both constraining and diversifying forces shape the viral genome, and these forces are also interrelated. We therefore built multivariate models using variables from both classes of forces. The statistical results are shown in Figure 2 and in Additional file 1, **Table S1**.

Conservation was governed by structured RNA (OR 2.09 (1.43-3.12), p = 0.0002) and domains located in α-helices (OR 1.57 (1.19-2.07), p = 0.001). On the other hand, flexible RNA regions (OR 0.68 (0.47-1.01), p = 0.05) and residues within CD4 T cell epitopes (OR 0.76 (0.6-0.97), p = 0.03) and antibody epitopes (OR 0.36 (0.26-0.5), p = 8.29E-10) tolerated more variability. A second model was built, excluding protein structure data, since this information is not available for the complete genome. In this second model, 2 additional variables were retained: overlapping reading frames associated with more variability (OR 0.51 (0.42-0.63), p = 1.33E-10), while residues within CD8 T cell epitopes associated with conservation (OR 1.24 (1.02-1.52), p = 0.03), Figure 2A.

We also performed this analysis for each gene separately (Additional file 1, **Table S1**). Conservation was associated with RNA structure in *RT*, *gp120 *and *gp41*. CD8 T cell epitopes in *tat *and *gp120 *were associated with increased variability. We also confirmed the association of variability with flexible RNA regions in *gp120*, with CD4 T cell epitopes in *gp120 *and with antibody epitopes in *tat *and *gp120*.

Similarly, we performed a multivariate analysis for the prediction of positive selection. Genome-wide, we found less positive selection in structured RNA regions (OR 0.3 (0.14-0.58), p = 0.001) and in α-helix domains (OR 0.65 (0.42-1), p = 0.05). This is consistent with the increased conservation observed in these regions. On the other hand, we found an increased number of residues under positive selection in overlapping regions (OR 7.75 (1.08-37.18), p = 0.02), in CD4 T cell epitopes (OR 1.76 (1.23-2.52), p = 0.002) and in antibody epitopes (OR 3.5 (2.28-5.37), p = 1.26E-08), also consistent with the significantly increased variability in these domains **(**Figure 2B). As an additional layer of variation, we looked at sites enriched in AG and AA dinucleotide motives in a genome-wide positional screen. The three defined patches (Figure 1) enriched in AG and AA dinucleotide motives were associated with sites under positive selection (OR 2.12 (1.31-3.35), p = 0.002).

Individual gene analyses confirmed the enrichment of positively selected sites in *pro *and *tat*, in overlapping reading frames and in *gp120 *and *nef *antibody epitopes. Some genes did not follow the genome-wide trends; CD4 T cell epitopes were associated with conservation in *gag*, and CD8 T cell epitopes, with conservation in *gag, gp41 *and *nef*. Although CD8 T cell epitopes were not associated with positive selection genome-wide, we found opposing results for *tat *(having fewer residues under positive selection), and *gp120 *(increased number of residues under positive selection). Similar opposing results were obtained for CD4 T cell epitopes: when the gene analysis was completed, *tat *and *rev *had less positively selected residues, while *gp120 *and *gp41 *had more regions under positive selection.

The analyses could be biased if they included multiple consecutive strains from the same individual, or from transmission chains. Because of difficulties retrieving sequence information automatically, we chose to perform a sensitivity analysis that would exclude closely related strains. This sub-analysis excluded sequences with a similarity of more than 93%. We thus obtained a dataset of 242 sequences. The statistical results for this dataset were fully consistent with the complete dataset analysis. Conservation was still determined by structured RNA regions (OR 2.06 (1.43-3.05), p = 0.0002) and α-helix protein domains (OR 1.54 (1.18-2.02), p = 0.002), while flexible RNA structures (OR 0.64 (0.44-0.94), p = 0.02), CD4 T-cell epitopes (OR 0.78 (0.62-0.99, p = 0.04) and antibody epitopes (OR 0.35 (0.26-0.49), p < 0.0001) tolerate more variability. Leaving out protein information in the model retained 2 more variables: overlapping region associated with variability (OR 0.49 (0.4-0.6), p < 0.0001) and CD8 T-cell epitopes with conservation (OR 1.28 (1.05-1.56), p = 0.02). The analysis did not consider whether the sequences were from *in vitro *cultured isolates or from *in vivo *sources.

### Distribution of signals across the genome

We assessed whether conservation and positive selection signals were equally distributed across the genome. Conservation was 80% in the 5' half of the genome versus 55% in the 3' half of the genome (OR 0.29 (0.25-0.34), p < 0.0001). Correspondingly, positive selection was observed in 7% of residues in the 5' half of the genome versus 18% in the 3' half of the genome (OR 0.32 (0.25-0.40), p < 0.0001).

All data used in this study, indexed to the HXB2 reference sequence, are available from the authors upon request.

## Discussion

We have constructed an in-depth map of the HIV-1 genome that presents the landscape of genetic variation in the context of several levels of structural and immunological constraints. Over two-thirds of the viral genome and proteome are conserved. Conservation is strongly determined by RNA structure and, at the protein level, by the need to maintain α-helix domains. On the other hand, 12% of the genome are under positive selection, with an enrichment of sites observed in CD4 T cell and antibody epitopes. Previous studies advanced the understanding of protein [7] or RNA [10] structural constraints on viral genome diversity, or on immune selective pressures [11,12]. Here, we examined the viral genome under the paradigm of multiple layers of information.

Genomes of single-stranded RNA viruses contain important structures that support internal ribosome entry sites, packaging signals, pseudoknots, transfer RNA mimics, ribosomal frameshift motifs, and *cis*-regulatory elements. Watts *et al*. [10] used high-throughput SHAPE to interrogate nucleotide flexibility in the HIV-1 genome, as well as estimates of pairing probability at each nucleotide. This approach led to the identification of 10 regions that exhibit both low SHAPE reactivity and high pairing probability. Most genome regions with low SHAPE reactivity were shown to associate with a regulatory function. They proposed a model in which, in addition to the linear relationship between RNA and protein primary sequences, there is a second level of higher order RNA structure that directly modulates ribosome elongation, thus influencing native protein folding and tertiary structure. Although the present study uses data on SHAPE reactivities derived from a single viral strain, Watts *et al*. [10] compared the empirical data with evolutionary base-pairing probabilities predicted using an alignment of non-recombinant group M subtype sequences from the Los Alamos database, and found that only four regions were in disagreement. Overall, the present study underscores that this novel component of the genetic code represents the strongest determinant of conservation.

Our study also indicates that protein structure, specifically α-helix domains, is associated with conservation. The α-helix is the most important and stable structural element in proteins [13]. In contrast, more variation can be accommodated by β-sheets. Importantly, both layers of constraint, RNA and protein structure independently determine conservation and limit viral escape from selective pressures. These results confirm findings by Sanjuan *et al*., describing an association between SHAPE reactivity and second-codon position diversity (as a measure for protein sequence variation) and non-synonymous substitution rates (as a measure for selective pressure) [14].

The non-conserved viral genome encompasses two classes of sites, variable residues under relaxing constrain, and sites that are identified as being under positive selection, indicating higher fitness in a given host environment. We investigated three canonical selective forces of adaptive immunity: CD8 T cell, CD4 T cell and antibody responses. The results identify pressures that reflect population effects; i.e., a number of hosts share both the selective factor (the host factor) and the direction of the selecting force (escape). Here, an association was established for CD4 T cell and antibody responses and positive selection at cognate epitopes. Escape from antibody responses has clearly been demonstrated to occur from the very earliest phases of HIV-1 infection [15,16]. Escape for CD4 T cell responses is far less clear cut; however, the relationship between CD4 T cell help and the maturation of the antibody response [17] may certainly contribute to the association with positive selection at cognate epitopes. However, the undisputed relevance of CTL action - widely associated with viral escape [16,18,19], was not identified at population level polymorphism. Our interpretation is that the diversity of restricting alleles in the human population [20], the large proportion of sites in the viral genome identified as coding for CD8 T cell epitopes, and the diversity of fitness consequences of escape at the different CD8 T cell epitopes, fail to create a local signature of positive selection that can be identified in the viral genome at the population level. In addition, Irausquin and colleagues [12] indicated that many nonsynonymous mutations in both CD8 T cell and CD4 T cell epitopes are subjected to conflicting evolutionary pressures, with positive selection favoring escape mutations within hosts expressing the respective presenting HLA molecule and purifying selection acting to remove them in the population at large. Another explanation could be that escape mutants without deleterious effects become quickly fixed in the population so that these epitopes are relatively conserved [11].

Overlapping reading frames are generally thought to be evolutionarily stable and to be conserved, as mutation in one frame can negatively affect the second gene [21]. However, we identified low conservation and positive selection in those regions. An important caveat, however, is that there are currently no methods available to identify reliably site-specific positive selective pressure in these regions [22]. Thus, the approach we used may overestimate positive selection in overlapping reading frames. We also considered a possible contribution of hypermutation to structural constraints and positive selection. Only three strains were identified as being hypermutated. The analysis also explored the distribution of APOBEC3G/F editing across the genome. Three patches showed enrichment in AG and AA dinucleotide motives in a genome-wide positional screen. Although they were associated with sites under positive selection, it is not possible to establish a link of causality between deamination and genome evolution.

The joint analysis of different sets of information generated a comprehensive view of the complete genome. However, gene-specific analyses showed instances of departure from the genome-wide estimates. For example, CD8 T cell epitopes were generally well conserved, except in *gp120*, where they were enriched for sites under positive selective pressure. Similarly, CD4 T cell epitopes were enriched for sites under positive selection genome-wide, but these epitopes were significantly more conserved in *gag*. Thus, the various constraints and selective pressures do not act evenly across the genome.

Overall, the present study extends previous analyses by using a larger curated dataset of near-complete subtype B genome sequences to jointly analyze different conservation and evolutionary forces, The study by Sanjuán *et al*. on the interplay between RNA structure and protein evolution [14] used a hundred sequences and excluded *tat *and *rev *from the analysis. The study by Irausquin *et al*., on T cell epitopes [12] used between 46 and 599 sequences (depending on the gene) and did not include *gp41 *and *gp120*; the genes with the strongest signals of positive selection in the present study. The study by Woo *et al*., [7] analyzed the relationship between protein structure and evolutionary pressures on HIV-1 *gag *and *env *proteins by studying solvent-accessibility as a measure for protein structure, and Shannon entropy as a measure for protein diversity. They found a clear relationship between dN/dS ratio and the solvent-accessibility of the residues in the protein, with surface amino acids being under positive selection, and buried amino acids under purifying selection. They found no relationship between variability (as measured by Shannon entropy) and protein structure (helix or strand). However, our results point to an association between conservation and domains in α-helices, but only in the genome-wide analysis. The recent identification of multidimensional constraints on HIV-1 Gag evolution [23] also points towards analyses that could benefit from the layers of information included in the current study with the goal of better identifying regions of immunological vulnerability.

## Conclusions

The global map of the viral genome can inform models on the possible evolutionary trajectories of the virus. It also identifies positively selected sites that are not in canonical CD8 T cell, CD4 T cell or antibody epitopes, indicating a class of residues that may represent unrecognized epitopes or that are subject of other host selective pressures, such as innate immune effectors. As an example, sequence adaptation has been observed in the viral capsid in rhesus macaques upon cross-species transmission of SIVsm, due to selective pressure provided by restrictive TRIM5 alleles [24]. Recently, Alter *et al*. [25] identified killer immunoglobulin-like receptors (KIR)-associated amino-acid polymorphisms in the HIV-1 sequence that indicate that KIR-positive natural killer cells can place immunological pressure on the virus. In addition to informing the combined analysis of host and viral genetic information [26], the results of this study may help reveal novel mechanisms of antiretroviral response.

## Methods

As of May 2011, 2723 full genome sequences were reported in the Los Alamos database http://www.hiv.lanl.gov/content/index, of which 1066 belonged to subtype B. We built a study dataset of 635 full genome subtype B sequences that were on average 9031 nucleotides long and had no large insertions upon visual inspection of the alignment in comparison to the reference sequence K03455. Viral subtype was re-evaluated using the online Rega HIV-1 Automated Subtyping Tool, which also scans for recombination http://jose.med.kuleuven.be/genotypetool/html/subtypinghiv.html. All sequences were subtype B, and none of them was recombinant. Nucleotide sequences where aligned based on the protein alignment using tranalign in the EMBOSS package http://emboss.sourceforge.net/ thereby assuring the correct reading frame. Alignments were manually curated using Genedoc, and stop codons were replaced by gaps http://www.psc.edu/biomed/genedoc. Variable regions were re-aligned manually in order to keep the reading frame intact. The final alignment, with the various genes in consecutive order resulted in a concatenated genome of 9282 nucleotides.

For the purpose of the study, a working definition of a conserved residue meant that more than 95% of the sequences harbor the same amino acid at a given position. Selection was determined using the single-likelihood ancestor counting (SLAC) method implemented in HYPHY http://www.datam0nk3y.org/hyphy/doku.php. The SLAC method in HYPHY estimates the number of nonsynonymous and synonymous changes that have occurred at each codon throughout evolution based on ancestral reconstruction. Significance is given by the p-value, comparing the likelihood of positive selection by the likelihood of synonymous mutation by chance. We determined the best nucleotide substitution model for this dataset (model 012234, meaning that meaning that all nucleotide substitutions had different rates except for A to T and C to G substitutions which had equal rates), and crossed this with the MG94 model to obtain a codon substitution model. RNA structure data were obtained from Watts *et al*. [10]. Protein structure data were inferred based on entries in the RCSB Protein Data Bank http://www.rcsb.org/pdb/home/home.do. CD8 T cell, CD4 T cell and antibody human epitope positions in HIV-1 subtype B were based on the epitope summary tables in the HIV Immunology database provided on the Los Alamos National Laboratory website http://www.hiv.lanl.gov/content/immunology/products.html. For CD8 T cell epitopes, we used the A list, that represents the best-defined HIV CTL/CD8 epitopes as described by Llano *et al *[27]. The sequences were screened for hypermutation using the online hypermut tool form Los Alamos http://www.hiv.lanl.gov/content/sequence/HYPERMUT/hypermut.html. Since only 3 sequences were classified as hypermutated, we did not remove them from the alignment. We also performed a genome-wide sliding window-based positional screen for mutated motives in the alignment as described in Suspène *et al*. [28], using a window size of 600, displaced at a 60 nucleotide interval. We considered positions for which the product/substrate ratio was more than 2.087 to be enriched in AG and AA dinucleotide motives. This arbitrary cutoff corresponds to the 95 percentile distribution of product/substrate ratio for the 635 genomes in the study.

Positive selections, inclusion in an epitope and location in an overlapping coding frame, were coded as binary variables. RNA structure determination was based on the SHAPE parameter (selective 2'-hydroxyl acylation analyzed by primer extension) in the paper by Watts *et al*. [10]: structured (SHAPE parameter < 0.25), random (0.25 ≥ SHAPE ≤ 0.5) or flexible (SHAPE > 0.5). An amino acid was considered part of a structured region if it belonged to an α-helix or a β-sheet, which were treated as separate variables for analysis. Fisher exact test was used for univariate analysis, and either logistic regression or binary Firth's penalized-likelihood logistic regression was used in multivariate analysis. Statistical analyses were performed in R version 2.13.0 http://www.r-project.org/.

## Competing interests

The authors declare that they have no competing interests.

## Authors' contributions

AT conceived the study; JS, JF and AT designed the study; JS, JF and IB analysed the data; DCD provided a significant intellectual contribution. All authors were involved in drafting this paper; all authors have read and approved the final manuscript.

## Supplementary Material

Additional file 1 **Table S1**. Results of the multivariate statistics for association with conservation or positive selection. Click here for file
