# Supplementary material for: Mapping of positive selection sites in the HIV-1 genome in the context of RNA and protein structural constraints
Source: Retrovirology. 2011 Nov 1;8:87. doi: 10.1186/1742-4690-8-87 (PMC3229471; doi:10.1186/1742-4690-8-87)
Supplement: Additional file 1 — Table S1. Results of the multivariate statistics for association with conservation or positive selection. [file 1742-4690-8-87-S1.DOCX]

**Supplementary Table S1**: Result of the multivariate statistics for association with (A) conservation, or (B) positive selection. The odds ratio (95% confidence interval) and the p-value are shown for the entire genome and for each gene separately. Results in bold are significant. NS: not significant, na: not applicable

* Variable that was significant when the protein structure was excluded of the multivariate model

| (A) Multivariate – conservation | | | | | | | | | | | | | | | | | | | | | | | | | | | | | | | | | | | | | | | | | | | | | | |
| --- | --- | --- | --- | --- | --- | --- | --- | --- | --- | --- | --- | --- | --- | --- | --- | --- | --- | --- | --- | --- | --- | --- | --- | --- | --- | --- | --- | --- | --- | --- | --- | --- | --- | --- | --- | --- | --- | --- | --- | --- | --- | --- | --- | --- | --- | --- |
|  | **Genome** | | **gag** | | **pro** | | | **rt** | | | | **int** | | | | **vif** | | | | **vpr** | | | **tat** | | | | **Rev** | | | | **Vpu** | | | | **gp120** | | | | **gp41** | | | | | **nef** | | |
|  | OR (95% CI) | P | OR (95% CI) | p | OR (95% CI) | p | | OR (95% CI) | | p | | OR (95% CI) | | p | | OR (95% CI) | | p | | OR (95% CI) | | p | OR (95% CI) | | p | | OR (95% CI) | | p | | OR (95% CI) | | p | | OR (95% CI) | | p | | OR (95% CI) | | p | | | OR (95% CI) | p | |
| Flexible RNA regions | **0.68 (0.47-1.01)** | **0.05** |  | na |  | na | | 1.33 (0.57-3.69) | | NS | | 1.32 (0.54-3.75) | | NS | | 0.87 (0.25-3.5) | | NS | |  | | na | 0.97 (0.0006-23.04) | | NS | | 0.28 (0.002-5.48) | | NS | | 0.35 (0.11-1.08) | | NS | | **0.43 (0.2-0.89)** | | **0.02** | |  | | na | | | 0.93 (0.12-5.94) | NS | |
| structured RNA regions | **2.09 (1.43-3.12)** | **0.0002** | 0.92 (0.35-2.91) | NS | 1.77 (0.41-12.36) | NS | | **3.3 (1.49-8.8)** | | **0.007** | |  | | na | | 0.63 (0.25-1.62) | | NS | | 1.14 (0.32-4.25) | | NS | 0.7 (0.033-107.2) | | NS | | 0.84 (0.14-4.59) | | NS | | 1.29 (0.063-194.7) | | NS | | **2.12 (1-4.66)** | | **0.05** | | na **(2.14 (1.19-3.92))*** | | na **(0.01)*** | | | 1.05 (0.56-1.97) | NS | |
| α-helix structures | **1.57 (1.19-2.07)** | **0.001** | 1.69 (0.96-2.96) | NS | 1.07 (0.13-22.76) | NS | | 1.02 (0.6-1.74) | | NS | | 0.83 (0.38-1.77) | | NS | |  | | na | |  | | na |  | | na | |  | | na | |  | | na | | 0.61 (0.3-1.26) | | NS | | 5.17 (0.54-45.73) | | NS | | |  | na | |
| β-sheet structures | 0.84 (0.63-1.12) | NS |  | na | 0.39 (0.11-1.19) | NS | | 1.31 (0.72-2.5) | | NS | | 1.93 (0.7-6.23) | | NS | |  | | na | |  | | na |  | | na | |  | | na | |  | | na | | 1.11 (0.67-1.86) | | NS | |  | | na | | |  | na | |
| Overlap-ping regions | 0.38 (0.12-1.47) **(0.51 (0.42-0.63))*** | NS (**1.33E-10**)* |  | na | **0.1 (0.007-0.97)** | **0.06** | |  | | na | |  | | na | | 1.46 (0.68-3.31) | | NS | | 0.79 (0.18-3.12) | | NS | **5.86 (2.26-16.26)** | | **0.0002** | |  | | na | | 0.68 (0.25-1.86) | | NS | |  | | na | |  | | na | | |  | na | |
| CD8 T cell epitope | 1.12 (0.84-1.52) **(1.24 (1.02-1.52))*** | NS (**0.03**)* | 1.28 (0.72-2.34) **(1.85 (1.11-3.14))*** | NS (**0.02**)* | 10.3 (0.78-299.8) | NS | | 0.9 (0.53-1.58) | | NS | | 5.55E+06(1.05E-44-∞) | | NS | | 1.2 (0.32-5.77) | | NS | | 0.36 (0.078-1.71) | | NS | **0.11 (0.017-0.53)** | | **0.005** | | 2.11 (0.77-6.11) | | NS | |  | | na | | **0.37 (0.15-0.81)** | | **0.02** | | 0.47 (0.076-2.98) **(1.78 (1.03-3.15))*** | | NS **(0.04)*** | | | **3.39 (1.83-6.45)** | **0.0001** | |
| CD4 T cell epitope | **0.76 (0.6-0.97)** | **0.03** | **3.02 (1.57-5.76)** | **0.0008** |  | na | | 1.05 (0.5-2.45) | | NS | | 1.84 (0.81-4.76) | | NS | |  | | na | | 1.08 (0.24-4.37) | | NS | 0.46 (0.18-1.16) | | NS | | 0.48 (0.22-1.01) | | NS | |  | | na | | **0.55 (0.35-0.89)** | | **0.02** | | 0.9 (0.13-7.92) | | NS | | | 1.04 (0.54-1.97) | NS | |
| AB epitope | **0.36 (0.26-0.5)** | **8.29E-10** | 3.58 (0.63-68.37) | NS |  | na | | 0.93 (0.37-2.68) | | NS | |  | | na | |  | | na | |  | | na | **0.17 (0.042-0.64)** | | **0.008** | |  | | na | |  | | na | | **0.54 (0.32-0.92)** | | **0.02** | | 1.81 (0.2-14.59) | | NS | | | 0.81 (0.19-4.17) | NS | |
|  | | | | | | | | | | | | | | | | | | | | | | | | | | | | | | | | | | | | | | | | | | | | | | |
| (B) multivariate – positive selection | | | | | | | | | | | | | | | | | | | | | | | | | | | | | | | | | | | | | | | | | | | | | | |
|  | **Genome** | | **Gag/** | | **Pro/** | | | | **rt** | | | **int** | | | **vif** | | | | **Vpr/** | | | | | **tat/** | | | | **Rev/** | | | | **Vpu/** | | | | **gp120** | | | | **gp41/** | | | **Nef/** | | | |
|  | OR (95% CI) | P | OR (95% CI) | p | OR (95% CI) | | p | | OR (95% CI) | | p | OR (95% CI) | p | | OR (95% CI) | | p | | OR (95% CI) | | p | | | OR (95% CI) | | p | | OR (95% CI) | | p | | OR (95% CI) | | p | | OR (95% CI) | | p | | OR (95% CI) | | p | OR (95% CI) | | | p |
| Flexible RNA regions | 0.94 (0.5-1.68) | NS |  | na |  | | na | | 1.1 (0.17-4.15) | | NS | 0.93 (0.14-3.91) | NS | | 0.55 (0.03-3.1) | | NS | |  | | na | | | 2.81 (0.017-80.7) | | NS | | 0.60 (0.004-11.83) | | NS | | 0.5 (0.087-2.04) | | NS | | 0.64 (0.27-1.38) | | NS | |  | | na | 0.5 (0.0037-5.06) | | | NS |
| structured RNA regions | **0.3 (0.14-0.58)** | **0.001** | 0.21 (0.001-1.67) | NS | 0.54 (0.004-6.03) | | NS | | 0.24 (0.013-1.18) | | NS |  | na | | 0.93 (0.2-3.1) | | NS | | 1.46 (0.21-10.31) | | NS | | | 1.93 (0.09-291.9) | | NS | | 1.91 (0.2-15.66) | | NS | | 1.1 (0.007-22.69) | | NS | | 0.45 (0.14-1.21) | | NS | |  | | na | 1.06 (0.41-2.63) | | | NS |
| α-helix structures | **0.65 (0.42-1)** | **0.05** | 0.72 (0.32-1.64) | NS | 2.91 (0.22-30.7) | | NS | | 1.47 (0.54-3.97) | | NS | 0.85 (0.22-3.26) | NS | |  | | na | |  | | na | | |  | | na | |  | | na | |  | | na | | 1.07 (0.42-2.52) | | NS | | 0.2 (0.001-38.96) | | NS |  | | | na |
| β-sheet structures | 0.98 (0.63-1.5) | NS |  | na | 0.88 (0.14-5.96) | | NS | | 1.34 (0.44-3.86) | | NS | 0.32 (0.017-2.12) | NS | |  | | na | |  | | na | | |  | | na | |  | | na | |  | | na | | 0.58 (0.32-1.07) | | NS | |  | | na |  | | | na |
| Overlap-ping regions | **7.75 (1.08-37.18)** | **0.02** |  | na | **20.79 (1.36-3104.4)** | | **0.03** | |  | | na |  | na | | 1.03 (0.35-2.68) | | NS | | 1.41 (0.35-6.68) | | NS | | | **11.11 (3.07-60.16)** | | **8.32E-05** | |  | | na | | 0.98 (0.3-3.06) | | NS | |  | | na | |  | | na |  | | | na |
| CD8 T cell epitope | 0.83 (0.52-1.29) | NS | 0.72 (0.29-1.68) | NS | 0.075 (0.0005-1.24) | | NS | | 0.6 (0.17-1.73) | | NS | 2.28 (0.1-21.68) | NS | | 1.37 (0.2-5.95) | | NS | | 0.45 (0.003-4.79) | | NS | | | **0.17 (0.016-0.95)** | | **0.04** | | 1.44 (0.35-5.59) | | NS | |  | | na | | 1.77 (0.8-3.79)  **(2.36 (1.31-4.17))*** | | NS **(0.003)*** | | 2 (0.21-23.8) | | NS | 0.67 (0.25-1.69) | | | NS |
| CD4 T cell epitope | **1.76 (1.23-2.52)** | **0.002** | 0.58 (0.24-1.53) | NS |  | | na | | 0.95 (0.14-3.6) | | NS | 1.12 (0.23-4.18) | NS | |  | | na | | 0.39 (0.06-2.28) | | NS | | | **0.28 (0.088-0.83)** | | **0.02** | | **0.093 (0.019-0.3)** | | **1.37E-05** | |  | | na | | 1.5 (0.87-2.61)  **(1.67 (1.06-2.63))*** | | NS **(0.03)*** | | **13.18 (1.11-1836.63)** | | **0.004** | 0.61 (0.25-1.51) | | | NS |
| AB epitope | **3.5 (2.28-5.37))** | **1.26E-08** | 0.41 (0.003-3.54) | NS |  | | na | | 0.64 (0.034-3.59) | | NS |  | na | |  | | na | |  | | na | | | 0.27 (0.046-1.13) | | NS | |  | | na | |  | | na | | **2.34 (1.3-4.27)** | | **0.004** | | 0.45 (0.022-87.12) | | NS | **5.74 (1.1-27.8)** | | | **0.04** |
